# The Effect of a Yoga‐Based Stress Management Program on Depression, Anxiety, Stress and Psychological Well‐Being in Adolescents: Cluster Randomized Controlled Trial

**DOI:** 10.1002/jad.70155

**Published:** 2026-04-12

**Authors:** Nesrin Arslan, Aysun Ardic

**Affiliations:** ^1^ Department of Nursing, Faculty of Health Sciences Karabuk University Karabük Turkey; ^2^ Florence Nightingale Faculty of Nursing Istanbul University‐Cerrahpasa Istanbul Turkey

**Keywords:** adolescent, anxiety, depression, school health, stress management, yoga

## Abstract

**Introduction:**

Globally increasing stress levels among adolescents pose a significant public health concern. Yoga‐based interventions are gaining attention as effective and holistic approaches to mental health. This study aimed to examine the effects of a Yoga‐Based Stress Management Program on adolescents' levels of perceived stress, anxiety, depression, and psychological well‐being.

**Methods:**

A cluster randomized controlled trial was conducted with 120 adolescents mean age = 16.9 ± 0.9 years; 77.5% female from eight high schools. The intervention group participated in a 4‐week program consisting of weekly 50‐min sessions integrating cognitive‐behavioral stress management with Yin yoga practice, while the control group received a minimal‐intervention condition (one‐time brief stress education session). Outcomes were assessed at baseline, post‐intervention, and at 3‐month follow‐up. Effect sizes are reported using partial eta squared (ηp²).

**Results:**

Significant group × time interaction effects were found for all outcomes, with large effect sizes observed for depression (ηp² = 0.651), anxiety (ηp² = 0.734), perceived stress (ηp² = 0.753), psychological well‐being (ηp² = 0.668), and yoga self‐efficacy (ηp² = 0.759). Improvements in the intervention group were evident post‐intervention and were largely maintained at follow‐up.

**Conclusion:**

The Yoga‐Based Stress Management Program was associated with meaningful improvements in adolescents' mental health and well‐being. These findings suggest that integrating stress management with yoga may represent a promising school‐based approach to support adolescent well‐being, although further studies with active control conditions are warranted.

## 1 Introduction

Adolescence is a stage of life shaped by both social and biological changes. Good and bad experiences in this period leave lasting traces on individuals (Janjhua et al. 2020). Biological changes in adolescence may weaken the mechanisms of coping with stress and pave the way for unhealthy behaviors (Bazzano et al. 2022). In addition, factors such as low self‐control and awareness affect mental health adversely (Parajuli et al. 2023). This increases the stress, anxiety, and depression levels of adolescents (Janjhua et al. 2020).

According to the World Health Organization data, it is estimated that 3.6% of adolescents between the ages of 10–14 experience anxiety and 1.1% experience depression, and of those aged 15–19 years, 4.6% experience anxiety and 2.8% depression (World Health Organization 2019). In a systematic review of 42 studies conducted in the USA, it was found that 31.5% of young people aged 11–18 years experienced depression (Feiss et al. 2019). Frustration, problems with peers, death of a loved one, high expectations, divorce of parents, and test anxiety can cause stress in adolescents (American Academy of Child and Adolescent Psychiatry 2021). In addition, stress affects academic achievement and school attendance adversely by disrupting health and well‐being (Bernal‐Morales et al. 2015; World Health Organization 2019). A study by the Organization for Economic Cooperation and Development (OECD) covering students aged 15–16 from 72 countries indicated that 66% of the students felt stressed about low grades, and 59% were worried about difficult exams (OECD 2017). Mental disorders such as depression and anxiety are associated with suicide, and suicide is one of the leading causes of death among 15–19‐year‐old individuals (World Health Organization 2019). Evidence suggests that experiencing stressful life events increases the risk of health problems, such as unhealthy living habits (Pascoe et al. 2020), cancer (Kruk et al. 2019), and heart diseases (Song et al. 2019). Therefore, it is vital to strengthen mental health via effective stress management.

### 1.1 Background: Yoga‐Based Stress Management and Adolescent Mental Health

Studies have proven that physical activity contributes to the improvement of mental health by affecting neurocognitive functions (Ardic and Erdogan 2017; Hoying et al. 2016; Radovic et al. 2017). Evidence suggests that the mental health effects of physical activity vary substantially depending on the type, intensity, duration, and contextual characteristics of the activity, and not all forms of physical activity exert the same psychological benefits (Vella et al. 2023). Yoga, which is a holistic movement practice that combines meditation, breathing exercises, and physical movements, is increasingly used in physical activity and mental health research (Vergeer and Biddle 2021). Unlike traditional physical activity interventions that primarily focus on aerobic intensity and energy expenditure, yoga represents an innovative mind‐body approach that integrates physical postures with breath regulation and attentional control (La Forge 2016; Vergeer and Biddle 2021). Adolescence is a developmental period characterized by heightened emotional reactivity and ongoing maturation of cognitive and emotional regulatory systems, which may increase vulnerability to stress, anxiety, and depressive symptoms (Parajuli et al. 2023). The number of people who prefer yoga, which is one of the innovative practices, instead of physical activity, is gradually increasing, especially among young people (Black et al. 2018). Although yoga is based on physical postures, it provides both physical and mental benefits (James‐Palmer et al. 2020). The postures in yoga, unlike a standard exercise (Felver et al. 2015; Karaguzel and Yenel 2019), are performed with breath and body awareness without mental or physical exertion (Birdee et al. 2015; Karagüzel and Yenel 2019). Some studies have shown a significant reduction in depression, anger, and negative affect (Felver et al. 2015) and a greater increase in mindfulness predicting stress reduction (Tong et al. 2021) in adolescents who practice yoga than physical activity. Mind‐body interventions such as yoga may be particularly suitable for adolescents, as they emphasize self‐awareness rather than performance or competition and can be easily implemented in school settings with minimal equipment (Sapthiang et al. 2019).

Yoga, a very old practice originating from India, is a mindfulness‐based practice that integrates mind and body with an inner focus beyond the usual body movements (La Forge 2016; Patanjali 2012; Parajuli et al. 2023). Yoga regulates sympathetic activity by bringing physical activity and relaxation techniques together (Kurwale and Gadkari 2014; Patanjali 2012) and improves emotion regulation skills and awareness (Parajuli et al. 2023). Unlike the commonly used styles of yoga, such as Hatha, Vinyasa, and Ashtanga, Yin yoga is a slow form of practice that involves standing still for more than a few minutes. While the body is engaged in static postures in Yin yoga, proper use of the breath provides space for bodily and mental self‐awareness. This allows the management of stress (Hylander et al. 2017). From a psychophysiological perspective, yoga practices have been shown to influence autonomic nervous system functioning by reducing sympathetic activation and promoting parasympathetic activity, which may contribute to reductions in physiological arousal and stress‐related symptoms. Through the integration of controlled breathing, sustained postures, and mindful attention, yoga may support both psychological and physiological pathways underlying stress regulation and emotional balance (Khajuria et al. 2023; Voss et al. 2023). Despite growing evidence for yoga‐based interventions in adolescent mental health, most school‐based studies have focused on dynamic yoga styles, and empirical evidence regarding slower, introspective practices such as Yin yoga in adolescent populations remains limited (Lin and Zhao 2025; Zoogman et al. 2019). In a systematic review, it was found in 58% of the studies reviewed that yoga was effective in improving mental health in adolescents, reducing both anxiety and depression levels (James‐Palmer et al. 2020). A meta‐analysis of yoga‐based studies on the evaluation of the effect of yoga on anxiety indicated that yoga significantly reduced anxiety symptoms, while more globally it had a curative effect on psychological symptoms (Zoogman et al. 2019). Yoga is recommended as a relaxing tool to reduce stress, especially for test anxiety (D'souza et al. 2021; Pascoe et al. 2020). Interventional studies to improve mental health in adolescents have mostly included different yoga styles other than Yin yoga (Bazzano et al. 2022; Felver et al. 2015; Hospital et al. 2023; Saxena et al. 2020).

Given the increasing mental health problems in adolescents (World Health Organization 2019), greater efforts are needed to prevent depression, anxiety, and stress and to improve psychological well‐being. In recent years, there has been an increasing interest in programs that aim to build resilience and increase protective factors in adolescents (Sapthiang et al. 2019). In this study, the stress management sessions of the COPE‐Health TEEN program and Yin yoga were used together to help adolescents gain stress management skills. In this way, it was aimed to create stronger ways to cope with stress. This study was conducted to examine the effect of the Yoga‐Based Stress Management Program on perceived stress, depression, anxiety, psychological well‐being, and yoga self‐efficacy levels in adolescents. Therefore, the present study aimed to examine the effects of a school‐based Yoga‐Based Stress Management Program integrating cognitive–behavioral stress management with Yin yoga practice on adolescents' mental health outcomes, within a developmental and preventive framework.

Based on previous research, it was hypothesized that adolescents participating in the Yoga‐Based Stress Management Program would demonstrate greater reductions in perceived stress, depression, and anxiety, as well as greater improvements in psychological well‐being and yoga self‐efficacy, compared with adolescents in the control group (Felver et al. 2015; James‐Palmer et al. 2020). The present study contributes to the existing literature by evaluating a school‐based intervention that integrates cognitive–behavioral stress management with Yin yoga, a slower and more introspective yoga style that has been underrepresented in adolescent mental health research. By adopting a developmental and preventive framework, this study provides novel evidence regarding the feasibility and potential benefits of mind–body interventions tailored to adolescents within real‐world school settings.

## 2 Methods

### 2.1 Design

A cluster‐randomized experimental study design with a pretest‐posttest control group and repeated measurements was used. Experimental and control groups were followed up before the intervention and in the 1st week and 3rd month after the intervention. Participants were not informed about group allocation, and outcome data were analyzed by a researcher blinded to intervention and control group assignments.

### 2.2 Hypothesis

1. The post‐intervention perceived stress, depression, and anxiety scores of the experimental group will be lower than those of the control group.

2. The post‐intervention psychological well‐being and yoga self‐efficacy scores of the experimental group will be higher than those of the control group.

### 2.3 Participants

The general population of the research consisted of 1200 high school senior students aged 16–18 in the Safranbolu district of Karabük province. Students in Safranbolu prepare for the university entrance exam in their last year of high school and go through a stressful period. For this reason, the research was conducted with high school senior students. Inclusion criteria for the study were being a high school senior student, having a high level of depression, anxiety, and stress, not having any physical or mental disability, and agreeing to participate in the research. Students who had practiced yoga before were not included in the study, considering it a potential effect on the power of the study. Levels of depression, anxiety, and perceived stress were assessed by the research team using validated self‐report screening instruments administered in the school setting. The study did not aim to establish clinical diagnoses; rather, it focused on identifying adolescents with elevated symptoms who could benefit from a school‐based preventive intervention. Therefore, no formal psychiatric interviews were conducted. Adolescents with self‐reported severe psychiatric conditions or physical disabilities that could interfere with participation were excluded based on school health records and parental reports. Given the school‐based and preventive nature of the intervention, comorbid conditions such as attention‐deficit/hyperactivity disorder (ADHD) were not used as exclusion criteria, which reflects real‐world school populations and should be considered when interpreting the findings.

There are 14 high schools in total in the Safranbolu district of Karabük province. However, the administrators of only eight schools agreed to participate in the study. The study population consisted of 750 12th‐grade adolescents in eight schools. Each school was designated as a “cluster.” Four of the schools were selected as experimental and four as control by using the simple random drawing method. In the first stage of the study, the perceived stress, anxiety, and depression levels of the adolescents were measured and a total of 250 students who met the research inclusion criteria were determined. Of these students, 130 were assigned to the intervention group and 120 to the control group.

**Figure 1:**
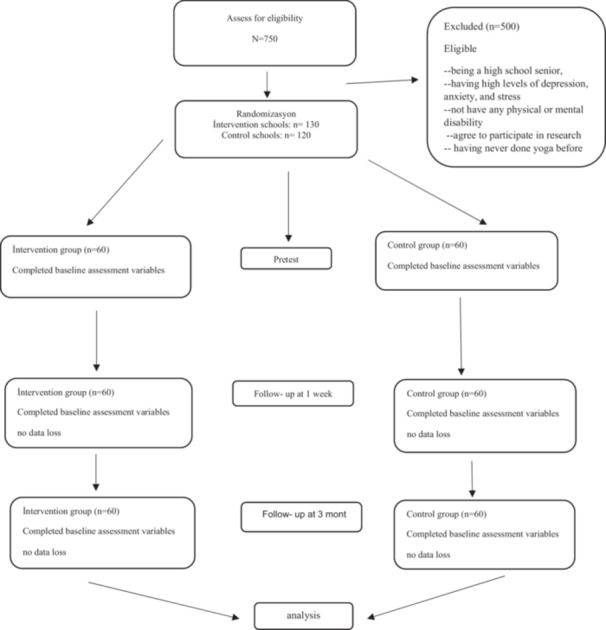
CONSORT followchart of the study.

For sample size estimation, previous school‐based intervention studies with repeated‐measures designs were used to inform the expected effect magnitude (Ardic and Erdogan 2017). Power considerations were based on a repeated‐measures ANOVA design (within‐between interaction) with two groups and three measurement points, with an alpha level of 0.05 and a target power of 0.95. Schools (as clusters) were randomly assigned to either the intervention group (*n* = 4 schools) or the control group (*n* = 4 schools) using a simple random method conducted by an independent researcher. Each school contributed students, selected based on inclusion criteria through a simple random sampling method. This cluster randomization approach was chosen to minimize contamination between groups and reflect the natural school‐based environment. Considering some attrition, a total of 120 students, including 60 in the intervention group and 60 in the control group, were included in the study. Randomization was conducted at the school (cluster) level to prevent contamination between students within the same school environment, while participant selection within each school was performed at the individual level. The participant flow through the study is presented in Figure 1 (CONSORT flow diagram).

### 2.4 The Intervention

#### 2.4.1 The Yoga‐Based Stress Management Program

The Yoga‐Based Stress Management Program consists of two parts, theoretical and practical. The program was implemented as 50‐min sessions once a week for 4 weeks. In the first 20 min of the sessions, theoretical issues related to stress management were shared. In the last 30 min of the program, Yin yoga was practiced with adolescents. The theoretical part of the program included the 4‐week‐long stress management sessions of the “Creating Opportunities for Personal Empowerment (COPE)‐Healthy Lifestyles Thinking, Emotions Exercise, and Nutrition (TEEN)” program (Table 1). The program had been developed by Melnyk et al. (2006) based on the Cognitive Behavioral Theory and Ecological Model (Melnyk et al. 2006). It was adapted to the Turkish language and culture by the researcher (Ardic and Erdogan 2017). The program teaches adolescents cognitive and behavioral skills and empowers them about their health. Thus, young people's skills of feeling positive, thinking positively, and behaving positively are developed, and it is ensured that they forget the bad experiences of the past and focus on the “present”/“the moment” (Ardic and Erdogan 2017; Melnyk et al. 2006).

**Table 1:** Structure and content of the Yoga‐Based Stress Management Program, including cognitive–behavioral stress management components and Yin yoga practices.

Session (20 min each)	Cope Health Teen Session Titles
1	Healthy lifestyles; Thinking, Feeling, Behavior Triangle/Focusing on the Moment
2	Self‐esteem and positive thinking
3	Goal setting and problem solving
4	Coping with stress and stress/Emotional and behavioral regulation
**Yin Yoga Flow (30 min)**	
2 min	Pranayama (participants were encouraged to observe the natural flow of their breath)
	***Asana***
4 min	Warm‐up
2 min	Malasana ‐ promotes grounding and release of physical tension.
2 min	Bhujangasana – supports spinal mobility and reduces stress‐related bodily stiffness.
3 min	Janu Sirsasana – encourages inward attention and calming of the nervous system.
3 min	Spinal twist – facilitates relaxation and gentle release of accumulated tension.
3 min	Ananda Balasana – promotes relaxation and parasympathetic activation.
3 min	Baddha Konasana – supports emotional release and relaxation.
3 min	Balasana – fosters a sense of safety and stress reduction.
5 min	Shavasana and focus on the moment for relaxing – facilitates deep relaxation and mindful breathing. (In this posture, participants were instructed to focus on their breathing.)
**Control grup 60 min**	short version of the stress sessions of the COPE Health TEEN program administered one time

*Note:* Each session included brief psychoeducation and cognitive–behavioral stress management strategies (e.g., identifying stressors, cognitive restructuring, relaxation techniques), followed by guided Yin yoga practice emphasizing slow‐paced postures, breath awareness, and mindful attention.

Yin yoga practice formed the intervention part of the program. Practices were carried out by the researcher, who was a yoga instructor and had ERYT‐200 and Yoga Alliance certificates. Yin yoga consists of postures that increase the flexibility of the connective tissue and the body. Following breathing exercises, yoga postures were taken. Adolescents were told to focus on the breath, mind, and body with a non‐judgmental awareness during the yoga flow.

Yin yoga practices took place in the indoor gymnasiums of the schools. Before the Yoga‐Based Stress Management Programme, a training booklet containing stress sessions and Yin yoga practice was distributed to the participants. They were given homework about the theoretical topic of each week and yoga. Program sessions and Yin yoga flow are presented in Table 1. The control group received a minimal‐intervention control condition consisting of a single 60‐min brief stress education session delivered once, without yoga practice or structured follow‐up sessions, and then continued their usual school routine.

### 2.5 Data Collection

This study was carried out in eight high schools in Safranbolu district of Karabük between April 2022 and August 2022. Four of the schools were determined as the experimental group and four as the control group. Data collection and intervention stages were carried out face to face. The intervention was performed 10 weeks before the university entrance test, which is associated with stress, and the last follow‐up was conducted 6 weeks after the test. The research design is presented in Figure 1. The variables in both groups were measured three times by the researchers before the intervention (T0) and in the first week (T1) and in the third month post‐interventionally (T2).

### 2.6 Measures

Study data were collected using a socio‐demographic form, created by the researchers, the Reynolds Adolescent Depression Scale, the Beck Anxiety Inventory, the Yoga Self‐Efficacy Scale, the Perceived Stress Scale, and the Psychological Well‐Being Scale.

#### 2.6.1 Sociodemographic Characteristics

To assess sociodemographic characteristics, a researcher‐developed form was used based on a review of the relevant literature. The form included questions on adolescents' age and gender, parental education levels, perceived quality of family and peer relationships, recent emotional experiences, and behavioral responses to stressful situations.

#### 2.6.2 Depressive Symptoms

To assess depressive symptoms, participants completed the Turkish version (Oskay 1997) of the Reynolds Adolescent Depression Scale (Reynolds 1986). Cronbach's alpha coefficient of the scale was 0.75. The scale consists of 30 items and has a 4‐point Likert‐type evaluation structure. Each item is scored between 1 and 4. Seven of the items (1, 5, 10, 12, 23, 25, and 29) are scored in reverse. Scores on the scale range from 30 to 120. High scores indicate high levels of depressive symptoms. It is recommended that adolescents who score ≥ 77 points on the scale should be evaluated more in terms of psychopathology. In this study, adolescents with a score of ≥ 77 were included in the sample. In this sample, Cronbach's alpha was found as 0.85.

#### 2.6.3 Anxiety Symptoms

To assess anxiety symptoms, participants completed the Turkish version (Ulusoy et al. 1998) of the Beck Anxiety Inventory (Beck et al. 1988). Cronbach's alpha value of the inventory, which consists of a total of 21 items that are scored between 0 and 3, was found as 0.93. High total scores indicate the severity of the anxiety experienced by the individual. The anxiety levels of patients are classified according to the scores on the inventory as follows: 0–7, low anxiety; 8–15, mild anxiety; 16–25, moderate anxiety; 26–63, high anxiety. In this sample, Cronbach's alpha was found to be 0.95. Students with an anxiety level of ≥ 16 points were included in the sample.

#### 2.6.4 Yoga Self‐Efficacy

To assess yoga self‐efficacy, participants completed the Turkish version (Tetik Kucukelci 2018) of the Yoga Self‐Efficacy Scale (Birdee et al. 2015) and Cronbach's alpha was found as 0.88. Each item on the scale is scored between 1 and 5, and there are no reverse items. The scale is used to measure the yoga self‐efficacy levels of individuals. High scores indicate a high level of self‐efficacy. In this sample, Cronbach's alpha coefficient was found as 0.80.

#### 2.6.5 Perceived Stress

To assess perceived stress, participants completed the Turkish version (Eskin et al. 2013), of the Perceived Stress Scale (Cohen et al. 1983) and the internal consistency coefficient was found a 0.84. The scale consists of 14 items and has a five‐point Likert‐type evaluation structure. Each item is rated from “never (0)” to “very often (4).” Seven items on the scale are reverse scored. A minimum of zero (0) and a maximum of 56 points are obtained from the scale. High scores indicate a high level of perceived stress. In this study, adolescents with a perceived stress level of ≥ 28 were included in the sample. Cronbach's alpha value of the scale was found to be 0.94.

#### 2.6.6 Psychological Well‐Being

To assess psychological well‐being, participants completed the Turkish version (Telef 2013) of the Psychological Well‐Being Scale (Diener et al. 2010). Cronbach's alpha coefficient of the scale was calculated as 0.80. Each item on the scale is scored from 1 to 7 points, with options ranging from “strongly disagree (1)” to “strongly agree (7).” All items are positive. The total scale score ranges from 8 to 56. A high score indicates that the person has psychological resources and power (Telef 2013). In this sample, Cronbach's alpha value of the scale was found to be 0.72.

### 2.7 Data Analysis

Statistical analyses were performed using SPSS® version 23.0 (IBM Corp., Armonk, NY, USA). Descriptive statistics were calculated as means and standard deviations for continuous variables and frequencies and percentages for categorical variables. Baseline group differences in demographic characteristics and outcome variables were examined using independent‐samples *t*‐tests for continuous variables and chi‐square or Fisher–Freeman–Halton exact tests for categorical variables. The level of statistical significance was set at *p* < 0.05.

To examine intervention effects over time, a mixed ANOVA with group × time interaction was used as the primary analysis. Both univariate (Greenhouse–Geisser corrected) results are reported. Effect sizes for F‐tests are presented as partial eta squared (ηp²), with values of 0.01, 0.06, and 0.14 indicating small, medium, and large effects, respectively (Richardson 2011). Assumptions of normality and sphericity were checked prior to analyses. Partial eta squared values may appear relatively large in repeated‐measures designs, particularly when intervention effects are strong and consistent across measurement points. In cluster randomized school‐based interventions with homogeneous baseline characteristics, within‐subject variance is reduced, which can result in higher estimates of ηp². Therefore, effect sizes should be interpreted in relation to the study design and intervention intensity rather than in isolation.

### 2.8 Ethical Statement

The institutional permission for the study was obtained from the Karabük Provincial Directorate of National Education (date: 2.14.2022, number: E‐44653020‐605.01‐42884553). Ethics committee approval was obtained from Karabük University Non‐Interventional Clinical Research Ethics Committee (date: 1.18.2022, number: 799). The students and their families were informed about the research and their written consent was obtained. Participants did not receive any financial support. The research was registered with the ID number “NCT05324709” obtained from clinicaltrials.gov.

## 3 Results

Of the 750 adolescents who agreed to participate in the study, 49.33% (*n* = 370) had high levels of perceived stress, 53.33% (*n* = 400) had high depression levels, and 50.66% (*n* = 380) had high levels of anxiety. Anxiety, depression, and perceived stress levels were found to be high in 33% of the adolescents (*n* = 250).

### 3.1 Baseline Characteristics and Comparisons of Intervention and Control Groups

According to the findings, 77.5% of the adolescents included in the study were female students and their mean age was 16.92 ± 0.87. Demographic characteristics of the intervention and control groups are given in Table 2. The groups were similar at baseline in terms of demographic and other baseline variables (*p* > 0.05) (Tables 2 and 3).

**Table 2:** Characteristics of adolescent baseline in the i̇ntervention and the control groups.

Characteristics	Intervention *n* = 60		Control *n* = 60		*p*	*Statistics*
	Mean	SD	Mean	SD		
**Age**	17.03	0.68	16.80	1.02	0.145	1.46^a^
	**n**	**%**	**n**	**%**		
**Sex**						
Famele	43	71.7	50	83.3	0.189	2.34^b^
Male	17	28.3	10	16.7		
**Family type**						
Immediate family	39	65	39	65	0.446	1.61^b^
Extended family	6	10	10	16.7		
Single parent home	15	25	11	18.3		
**Mother's education**						
≤ secondary school	25	41.6	31	51.6	0.673	2.58^b^
High school	28	46.7	22	36.7		
University and above	7	11.7	7	11.7		
**Father's education**						
≤ secondary school	25	41.7	30	50	0.669	2.58^b^
High school	23	38.3	18	30		
University and above	12	20	12	20		
**How they interprets their health**						
Good	19	31.7	18	30	0.980	0.04^b^
bad	36	61.7	37	61.7		
too bad	5	6.7	5	8.3		
**Family Relationship**						
Very good	3	5	2	3.3	0.754	1.46^c^
Good	29	48.3	25	41.7		
Bad	25	41.7	31	51.7		
Too bad	3	5	2	3.3		
**Friend Relationship**						
Very good	2	3.3	3	5.0	0.586	1.36^c^
Good	28	46.7	22	36.7		
Bad	30	50.0	35	58.3		
**How they felt recently**						
stressful	31	51.7	28	46.7	0.466	2.57^c^
disconcerting	14	23.3	11	18.3		
furious	2	3.3	6	10		
desperate	13	21.7	15	25		
**How they behaved in stressful situations.**						
breath work	3	5	2	3.3	0.915	1.71^c^
physical activity	11	18.3	11	18.4		
nothing	35	58.3	35	58.3		
to cry	4	6.7	6	10		
shout at someone	7	11.7	6	10		

*Note:* a, Independet *t* test; b, Chi‐square; c, Fisher freeman halton exact; there were no statistically significant differences between the groups at baseline (*p* > 0.05).

**Table 3:** Comparison of variables before the program.

	Intervention *n* = 60	Control *n* = 60	Statistics	
	Mean (SD)	Mean (SD)	*t*	*p*
**Variable**				
Anxiety	48.25 (4.38)	46.91 (4.56)	1.63	0.105
Psychological Well‐Being	23.38 (4.78)	24.15 (5.00)	−0.858	0.393
Yoga Self‐Efficacy	23.30 (3.90)	23.81 (5.88)	−0.530	0.597
Body	8.51 (1.26)	8.61 (1.47)	−0.398	0.691
Breath	8.91(2.06)	8.96 (2.34)	−0.124	0.901
Mind	5.86 (1.68)	6.38 (2.21)	−1.438	0.153
Perceived Stress	46.10 (4.76)	44.90 (4.20)	1.463	0.120
Depression	93.30 (7.24)	91.51 (7.75)	−1.301	0.196

*Note: t*, Independet *t* test; there were no statistically significant differences between the groups at baseline (*p* > 0.05).

### 3.2 Effects of the Yoga‐Based Stress Management Program on Adolescent's Variables

The mean and standard deviation values of adolescents' depression, anxiety, perceived stress, yoga self‐efficacy, and psychological well‐being are presented in Table 4. Mixed ANOVA revealed significant group × time interaction effects for all outcome variables.

**Table 4:** Effects of the program on outcome variables.

Variables	Groups	Baseline (T0) Mean (SD)	Post İntervention 1st Week (T1) Mean (SD)	3rd month (T2) Mean (SD)	*Statistics*			
					*F (GG)*	*ηp² (Time × *Groups*)*	*F (GG)*	*ηp² (Time)*
Anxiety	Intervention	48.25 (4.38)	22.96 (7.07)	10.81 (6.88)	**326.027**	**0.734** a ***p*** < **0.001** * T0 > T1, T2 T1 > T2	**351.153**	**0.748** a ***p*** < **0.001** *
	Control	46.91 (4.56)	45.90 (6.03)	46.36 (6.67)				
Psychological Well‐Being	Intervention	23.38 (4.78)	40. 35 (5.51)	49.66 (4.68)	**237.787**	**0.668** a ***p*** < **0.001** * T0 < T1,T2 T1 < T2	**347.387**	**0.746** a ***p*** < **0.001** *
	Control	24.15 (5.00)	30.56 (5.69)	25.75 (5.57)				
Yoga Self‐Efficacy	Intervention	23.30 (3.90)	44.11 (2.71)	51.83 (5.90)	**215.587**	**0.646** a ***p*** < **0.001** * T0 < T1,T2	**370.694**	**0.759** a ***p*** < **0.001** *
	Control	23.81 (5.88)	35.66 (6.45)	28.85 (6.16)				
Body	Intervention	8.51 (1.26)	15.70 (1.23)	18.41 (1.53)	**146.675**	**0.554** a ***p*** < **0.001** * T0 < T1,T2 T1 < T2	**251.577**	**0.681** a ***p*** < **0.001** *
	Control	8.61 (1.47)	10.31 (3.33)	9.70 (2.92)				
Breath	Intervention	8.91(2.06)	16.05 (1.57)^†^	19.28 (5.38)	**914.941**	**0.413** a ***p*** < **0.001** * T0 < T1,T2	**105.067**	**0.471** a ***p*** < **0.001** *
	Control	8.96 (2.34)	10.86 (3.27)	9.18 (2.43)				
Mind	Intervention	5.86 (1.68)	12.36 (0.84)	14.13 (0.98)	**176.568**	**0.599** a ***p*** < **0.001** * T0 < T1,T2	**417.165**	**0.780** a ***p*** < **0.001** *
	Control	6.38 (2.21)	9.75 (1.95)	7.54 (2.27)				
Depression	Intervention	93.30 (7.24)	63.68 (7.20)	45.30 (10.35)	**219.763**	**0.651** a ***p*** < **0.001** * T0 > T1, T2 T1 > T2	**334.465**	**0.739** a ***p*** < **0.001** *
	Control	91.51 (7.75)	82.70 (6.67)	87.35 (6.81)				
Perceived Stress	Intervention	46.10 (4.76)	16.10 (5.56)	11.00 (5.98)	**360.030**	**0.753** a ***p*** < **0.001** * T0 > T1, T2	**572.331**	**0.829** a ***p*** < **0.001** *
	Control	44.90 (4.20)	36.55 (4.02)	43.61 (4.01)				

* *p* < 0.05, F = A Mixed ANOVA.

^a^ eta squared large effects, GG: Greenhouse‐Geisser, Effect sizes are reported as partial eta squared (ηp²) for group × time interaction effects.

Significant group × time interaction effects were observed both at the post‐intervention assessment and at the 3‐month follow‐up. Specifically, adolescents in the intervention group showed significantly greater reductions in depression (F = 219.763, *p* < 0.001, ηp² = 0.651), anxiety (F = 326.027, *p* < 0.001, ηp² = 0.734), and perceived stress (F = 360.030, *p* < 0.001, ηp² = 0.753) compared with the control group. In addition, psychological well‐being (F = 237.787, *p* < 0.001, ηp² = 0.668) and yoga self‐efficacy (F = 215.587, *p* < 0.001, ηp² = 0.646) increased significantly in the intervention group relative to controls, with large interaction effect sizes.

**Figure 2:**
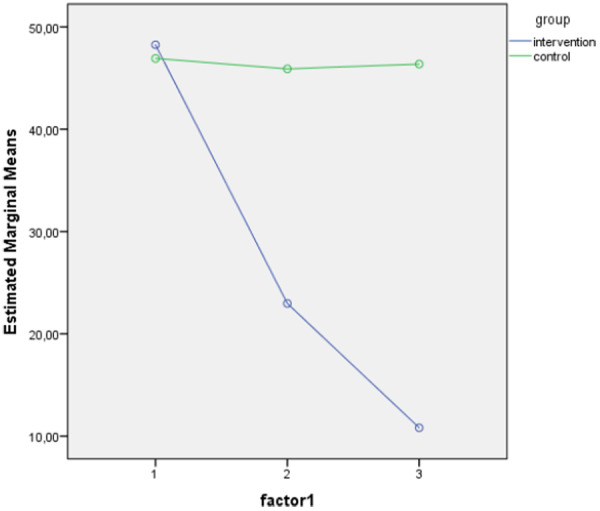
Anxiety.

**Figure 3:**
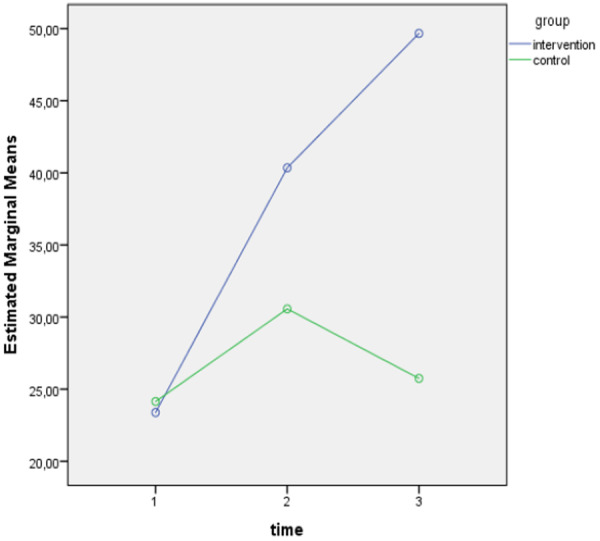
Psychological well‐being.

**Figure 4:**
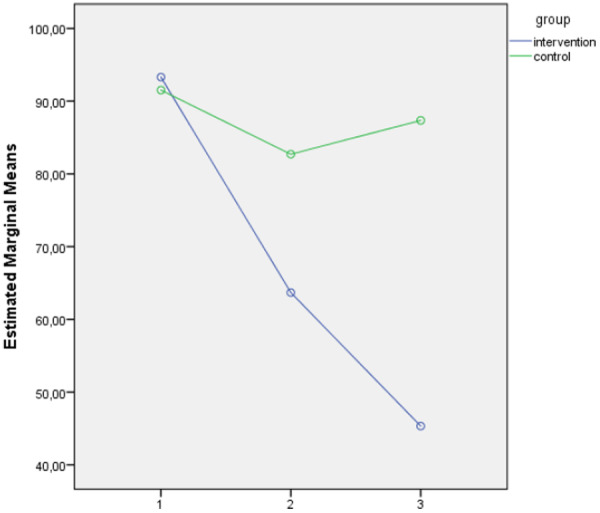
Depression.

**Figure 5:**
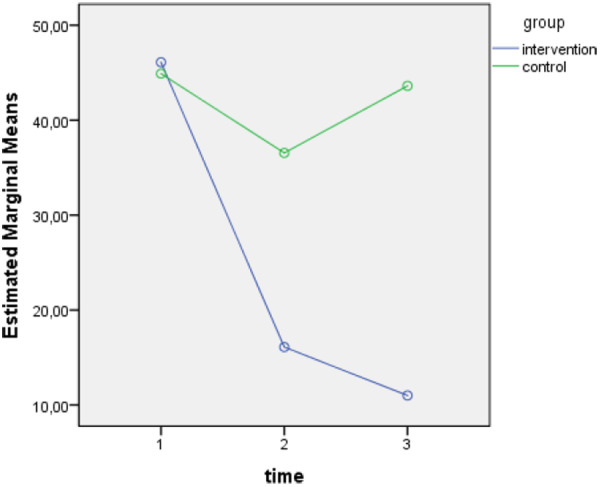
Perceived stress.

Post‐hoc analyses indicated that significant improvements in the intervention group occurred from baseline (T0) to post‐intervention (T1), and these effects were either maintained or further enhanced at the 3‐month follow‐up (T2). The reported partial eta squared values represent the overall group × time interaction effects across all measurement points rather than effects at a single time point (Table 4). The main outcome trajectories across measurement points are illustrated in Figures 2, 3, 4, 5.

## 4 Discussion

The present study examined the effects of a school‐based Yoga‐Based Stress Management Program on adolescents' mental health. Adolescents in the intervention group showed significantly greater improvements in depression, anxiety, perceived stress, and psychological well‐being compared with the control group, with effects sustained at the 3‐month follow‐up. These findings suggest that integrating stress management with Yin yoga may represent a promising approach to support adolescent well‐being, potentially through improvements in emotion regulation, attentional control, and psychophysiological calming mechanisms, as described in previous studies (Goldin et al. 2016; Zakiei et al. 2021). The further reduction in anxiety observed between the post‐intervention assessment and the 3‐month follow‐up may reflect sustained intervention effects. In particular, adolescents may have continued to apply the stress management techniques and yoga‐based breathing and awareness practices learned during the program in their daily lives. Such continued practice and skill consolidation over time may contribute to gradual improvements in anxiety beyond the formal intervention period.

In recent years, the rate of mental health problems in adolescents has been increasing and requires early intervention. Mental health promotion interventions should include strengthening individuals' capacity to regulate their emotions and building resilience to manage difficult situations and problems. Therefore, intervention programs should be conducted in settings that facilitate access to adolescents, such as schools or healthcare environments (World Health Organization 2019; van Loon et al. 2020).

Today, interest in yoga is increasing, especially in solving mental health problems including stress, depression, and anxiety (Bazzano et al. 2022; Janjhua et al. 2020). In this study, the Yoga‐Based Stress Management Program was used for the first time in the school environment for adolescents in Turkey. One of the most important goals of the COPE Health TEEN program used in the theoretical part of the study is to develop positive thinking, positive feelings, and positive behavior and includes physical activities (Ardic and Erdogan 2017). This program has been used in different studies in children and adolescents, and its effect on depression, anxiety, and stress levels was found in the range of weak to strong effect (Cohen's d: 0.37–1.81) at 0‐12 months follow‐up (Ardic and Erdogan 2017; Hoying et al. 2016; Melnyk et al. 2015). Unlike previous studies, this study used yoga practice instead of physical activity for the first time. The goal of ‘staying in the present moment' described in the stress sessions was integrated with Yin yoga. This practice aimed to achieve spiritual healing via physical and mental awareness. Unlike previous studies on the use of the COPE Health TEEN program, this study was found to have a large effect (0.413–0.753) for 3 months (Table 4). The comparison of the results suggested that the success of this effect was due to the practice of yoga.

In parallel with the current research, in studies conducted using various yoga styles, it was reported that adolescents' depression, anxiety, and stress levels decreased (D'souza et al. 2021; Hospital et al. 2023; Lemay et al. 2021; Sarkissian et al. 2018). Contrary to these results, two studies did not report a significant difference in the decrease in the stress, anxiety, and depression levels of adolescents following the yoga program (Bazzano et al. 2022; Saxena et al. 2020), whereas in one study, although no improvement was observed in adolescents' mental health problems, their self‐control and awareness levels were found to develop (Parajuli et al. 2023). Sarkissian et al. (2018) found that a 10‐week Kundalini yoga program improved students' stress with a moderate effect size. In the study of Lemay et al. (2021), Vinyasa yoga and meditation interventions were applied separately, a maximum of 30 min was given, and no homework was assigned. At the end of 6 weeks, it was determined that students' exam stress and anxiety had decreased. In another study, it was found that the Yoga Nidra intervention applied for 21 days reduced the level of academic stress in students aged 14–16 (D'souza et al. 2021). Hospital et al. (2023) reported that the anxiety and depression levels of participants decreased at the end of the 12‐week Vinyasa program. In another study conducted by Hylander et al. (2017) including a group with a mean age of 28 ± 11, 60 min of Yin yoga and 30 min of mindfulness practices were applied separately for 5 weeks, and it was found that the program had a moderate to large effect on reducing stress and anxiety and increasing awareness.

Self‐efficacy is the perception of the individual that affects their self‐realization, gaining positive health behaviors, and controlling health (Karadas 2022). Some studies suggest that a person's self‐efficacy may affect their attendance at yoga practices (Birdee et al. 2015) and stress control (Sarkissian et al. 2018). The integration of body, breath, and mental processes in yoga practice leads to happiness, peace, feeling good, and being healthy (Birdee et al. 2015; Tetik Kucukelci 2018). In the current study, yoga self‐efficacy was examined in body, breath, and mind domains, which reflect yoga. Following the intervention, yoga self‐efficacy in the yoga group increased over time and in a large effect size compared to the control group (Table 4). As a new finding, the increase in adolescents' yoga self‐efficacy levels was also found to be in line with the mental health results of the current study. This result suggested that the increase in yoga self‐efficacy affected the improvement in mental health. In conclusion, the findings of the study showed that the Yoga‐Based Stress Management Program has extremely positive effects on stress, depression, anxiety and well‐being in adolescents.

### 4.1 Implications for Practice

Yoga and yoga‐based practices have become increasingly popular in recent years. Considering this situation, yoga may be an interesting alternative for adolescents who are at risk for mental health. Implementation of yoga‐based programs in school environments in stress management not only attracts attention among adolescents but also has the potential to have widespread effects. The Yoga‐Based Stress Management Program can be used by health professionals and educators as a powerful intervention program for improving mental health in adolescents.

### 4.2 Strengths and Limitations

The strength of this research is that the sample was selected using a cluster randomized method and that stress management sessions were delivered using clearly defined, evidence‐based intervention methods, including yoga postures. None of the adolescents dropped out of the program, and no adverse events were reported during the study.

However, this study has several limitations. First, the results were based on adolescents' self‐reports. Second, six schools could not be included in the study because yoga was perceived as a religious ritual by school administrators, which may limit generalizability. Another important limitation is the use of a minimal‐intervention control condition. Although the control group received a single brief stress education session, this design does not allow full separation of specific and non‐specific intervention effects. Therefore, the observed effects cannot be fully attributed solely to the yoga practice itself, as factors such as increased attention from study staff, group interaction, or structured weekly activities may have contributed to the improvements observed in the intervention group.

Nevertheless, the use of a minimal‐intervention control condition reflects real‐world school settings, where implementing multiple parallel programs may not always be feasible, as also described in previous school‐based intervention studies (Eisenhut et al. 2022; Mousavi et al. 2023; Norouzi et al. 2020).

## 5 Conclusions

The Yoga‐Based Stress Management Program, carried out in the school environment, proved feasibility with positive results in adolescents' stress, anxiety, psychological well‐being, depression, and yoga self‐efficacy levels over time. However, for a sustainable impact, the program should be conducted in this age group with different cultures and religious beliefs, and in a larger sample. In future research, programs to reduce potential barriers to yoga interventions can be organized. There is also a need for research on the examination of participants' experiences in programs involving yoga interventions.

## Author Contributions

**Nesrin Arslan:** methodology, conceptualization, data curation, validation, supervision, funding acquisition, writing – original draft, writing – review and editing, investigation, formal analysis, software, visualization, resources. **Aysun Ardic:** supervision, software, methodology, investigation, funding acquisition, conceptualization, project administration.

## Funding

The authors have nothing to report.

## Ethics Statement

The research was registered with the ID number “NCT05324709” from clinicaltrials.gov. The institutional permission for the study was obtained from the Karabuk Provincial Directorate of National Education (date: 14.2.2022, number: E‐44653020‐605.01‐43482512). Ethics committee approval was obtained from Karabuk University Non‐Interventional Clinical Research Ethics Committee (date: 18.1.2022, number: 799). The author(s) agrees to take responsibility for ensuring that the choice of statistical approach is appropriate and is conducted and interpreted correctly as a condition to submit to the Journal.

## Conflicts of Interest

The authors declare no conflicts of interest.

## Data Availability

The authors have nothing to report.
